# Effects of N-Acetylcysteine and Glycine Administration on Serum Levels of Soluble Suppressor of Tumorigenicity 2 and Cardiac Morphological Characteristics in an Animal Model of Streptozotocin-Induced Diabetes

**DOI:** 10.3390/pathophysiology33040075

**Published:** 2026-10-07

**Authors:** Malik Ejubović, Radivoj Jadrić, Sabaheta Hasić, Emina Kiseljaković, Lejla Alić, Esad Ćosović, Dina Kapić, Orhan Lepara, Amira Jagodić Ejubović, Almir Fajkić

**Affiliations:** 1 Cantonal Hospital Zenica, Crkvice 67, 72000 Zenica, Bosnia and Herzegovina; amira_jagodic@hotmail.com; 2 Faculty of Medicine, University of Sarajevo, Čekaluša 90, 71000 Sarajevo, Bosnia and Herzegovina; radivoj.jadric@mf.unsa.ba (R.J.); sabaheta.hasic@mf.unsa.ba (S.H.); emina.kiseljakovic@mf.unsa.ba (E.K.); lejla.alic@mf.unsa.ba (L.A.); esad.cosovic@mf.unsa.ba (E.Ć.); dina.kapic@mf.unsa.ba (D.K.); orhan.lepara@mf.unsa.ba (O.L.); almir.fajkic@mf.unsa.ba (A.F.)

**Keywords:** diabetic cardiomyopathy, N-acetylcysteine, glycine, sST2, cardiac remodeling

## Abstract

**Background/Objectives:** Diabetic cardiomyopathy (DCM) is a major cardiovascular complication of diabetes mellitus characterized by progressive myocardial remodeling driven by oxidative stress, inflammation, and fibrosis. The interleukin-33 (IL-33)/soluble suppression of tumorigenicity 2 (sST2) signaling axis has recently emerged as an important regulator of cardiac remodeling and a potential therapeutic target in DCM. N-acetylcysteine (NAC) and glycine exhibit complementary antioxidant and anti-inflammatory properties; however, their combined effects on circulating IL-33 and sST2 levels and myocardial structural remodeling in experimental diabetes remain insufficiently investigated. This study evaluated the effects of NAC and glycine, administered alone or in combination, on oxidative stress, inflammatory biomarkers, cardiac morphology, and serum sST2 levels in streptozotocin-induced diabetic rats. **Methods:** Thirty-four adult Wistar rats were randomly assigned to five groups: healthy controls, untreated diabetic animals, diabetic animals treated with NAC (100 mg/kg/day), glycine (250 mg/kg/day), or combined NAC and glycine for 12 weeks. Diabetes was induced by a single intraperitoneal injection of streptozotocin (55 mg/kg). Serum concentrations of sST2, IL-33, malondialdehyde (MDA), and superoxide dismutase (SOD) activity were determined. Cardiac remodeling was evaluated by stereological and histopathological analyses. **Results**: Experimental diabetes significantly increased serum sST2, IL-33, and MDA concentrations and induced marked myocardial remodeling characterized by expansion of the interstitial and perivascular compartments, increased connective tissue deposition, and reduced cardiomyocyte volume density. Both NAC and glycine significantly attenuated these biochemical and structural abnormalities compared with untreated diabetic animals. Treatment effects varied across outcomes. The combined NAC and glycine regimen was associated with particularly pronounced reductions in sST2 and IL-33 and with preservation of myocardial architecture, whereas the magnitude of improvement in oxidative stress markers differed between treatment groups. **Conclusions:** NAC and glycine, administered either alone or in combination, attenuated several biochemical and structural alterations associated with streptozotocin-induced diabetes. The magnitude of these effects varied across the assessed outcomes, with the combined regimen showing particularly favorable effects on circulating sST2 and IL-33 levels and myocardial structural remodeling, while some oxidative stress parameters showed greater improvement with NAC monotherapy. These outcome-dependent effects highlight the potential complementary actions of NAC and glycine without implying uniform superiority of the combined regimen. Overall, these findings provide an experimental basis for further investigation of NAC- and glycine-based therapeutic approaches for diabetes-associated myocardial remodeling.

## 1. Introduction

Diabetic cardiomyopathy (DCM) is a special clinical condition marked by cardiac dysfunction in individuals with diabetes mellitus (DM), occurring without the presence of coronary artery disease, hypertension, or heart valve abnormalities. Chronic hyperglycemia generates various metabolic changes that significantly impact heart structure and function. One of the first signs of DCM is left ventricular hypertrophy and myocardial fibrosis, which results in impaired relaxation and diastolic dysfunction, subsequently followed by systolic impairment and visible heart failure with reduced ejection fraction [1,2,3].

Oxidative stress is a fundamental mechanism connecting diabetes to cardiovascular diseases. Continuously elevated blood sugar levels raise the levels of reactive oxygen species (ROS) through several associated mechanisms, such as glucose autoxidation, activation of the polyol pathway, or activation of protein kinase C (PKC) [4,5,6,7,8,9,10,11].

A surplus of reactive oxygen species (ROS) can overload the body’s natural antioxidant defenses, which can cause lipid peroxidation, protein modifications, and DNA damage. In a diabetic heart, increased fatty acid absorption and oxidation contribute to more prevalent mitochondrial ROS production, which causes mitochondrial dysfunction, the opening of permeability transition pores, and the initiation of apoptotic signaling [2,12]. These oxidative alterations hinder energy metabolism, contractile function, and calcium homeostasis, facilitating both diastolic and systolic failure. This creates an ongoing process in which oxidative stress causes metabolic inflexibility, damage to mitochondria, and changes in structure, all of which are evidence of DCM [13].

Inflammation seems to be a further contributor responsible for diabetes and related cardiovascular disorders. Obesity and long-term nutritional overload cause macrophages to gather and become active in metabolic tissues, such as adipose tissue, the liver, skeletal muscle, and pancreatic islets. This process results in the development of low-grade chronic inflammation [14,15,16,17,18]. Proinflammatory cytokines, including TNF-α, IL-1β, and IL-6, disrupt insulin signalling and promote systemic and cardiac insulin resistance by triggering serine phosphorylation of insulin receptor substrates through the c-Jun N-terminal kinase (JNK) inhibitor κB kinase/nuclear factor kappa B (IKK/NF-κB) pathways [15,18]. Insulin signalling is further inhibited by elevated IL-6 levels through JAK–STAT pathways, which further links inflammatory signalling to metabolic dysregulation. Chronic inflammation in the myocardium induces fibroblast activation, extracellular matrix remodelling, and endothelial dysfunction, which subsequently contribute to the structural and functional alterations that are suggestive of DCM [19].

Soluble suppression of tumorigenicity 2 (sST2) has become a significant predictive biomarker in cardiovascular disease and diabetes. sST2 constitutes a portion of the interleukin-1 receptor family and works as a decoy receptor for interleukin-33 (IL-33), which inhibits its cardioprotective effects. IL-33/ST2L signalling has effects on the heart that prevent hypertrophy, fibrosis, and inflammation. People with diabetes who have higher levels of sST2 in their blood are associated with more myocardial fibrosis, inadequate remodelling, and worse heart failure outcomes. sST2 indicates both mechanical cardiac stress and systemic inflammatory activity, including various pathogenic pathways associated with DCM. Its increase is associated with more severe illness, diastolic dysfunction, and a poorer prognosis, making it a valuable biomarker for identifying individuals at risk and monitoring treatment in those with diabetes and heart problems [20,21,22,23,24,25,26].

Given the central roles of oxidative stress and inflammation in diabetic cardiomyopathy, antioxidant strategies may offer a potential approach to attenuating diabetes-induced cardiac remodelling. We hypothesised that N-acetylcysteine (NAC) and glycine, particularly when administered in combination, would reduce oxidative and inflammatory disturbances and consequently mitigate myocardial structural alterations in streptozotocin-induced diabetic rats. Therefore, this study aimed to evaluate the effects of NAC and glycine, administered alone or in combination, on serum sST2 and IL-33 levels, oxidative stress markers, and cardiac morphological and stereological characteristics in experimental diabetes [1,2,3,22,23,24,25,26,27,28].

## 2. Materials and Methods

### 2.1. Experimental Design

The experimental procedures were carried out using 34 adult male albino Wistar rats, healthy at baseline prior to the experimental procedures, aged 10 weeks and weighing between 180 and 380 g. Type 1 diabetes mellitus was induced by a single intraperitoneal injection of STZ dissolved in citrate buffer (pH 4.5) at a dose of 55 mg/kg. Seventy-two hours after STZ administration, blood glucose concentrations were measured using a commercially available glucometer, and animals with blood glucose levels > 11.1 mmol/L were considered diabetic. Control animals received an equivalent volume of citrate buffer [29,30,31].

The animals were randomly allocated into five groups:

- Group 1—Control (8): Healthy rats with no pharmacological intervention, used to establish baseline cardiac morphological characteristics, serum sST2 levels, and inflammatory and oxidative stress parameters.
- Group 2—DM (8): Diabetic rats without therapeutic intervention, observed for up to 12 weeks, corresponding to the planned maximum duration of the experiment.
- Group 3—NAC (6): Diabetic rats received oral N-acetylcysteine (NAC) at 100 mg/kg via syringe seven days after diabetes confirmation, followed by daily administration in drinking water, and was observed for 12 weeks.
- Group 4—Glycine (6): Diabetic rats received oral glycine at 250 mg/kg starting seven days after diabetes confirmation, followed by daily administration in drinking water for 12 weeks.
- Group 5—NAC + Glycine (6): Diabetic rats received a combination of NAC (100 mg/kg) and glycine (250 mg/kg) orally seven days after diabetes confirmation, followed by daily administration for 12 weeks.

NAC and glycine were administered once daily at the predefined dose after dissolution in a small volume of water and direct oral administration. After dosing, animals had free access to untreated drinking water ad libitum.

For presentation in tables, the experimental groups were abbreviated as follows: Control, control group; DM, diabetes mellitus group; NAC, NAC-treated group; Glycine, glycine-treated group; and NAC + Glycine, combined-treatment group.

### 2.2. Sample Collection and Tissue Preparation

Twenty-four hours after the final treatment, following completion of the 12-week experimental period (day 85), animals were anaesthetised with ketamine and sacrificed using standard procedures in accordance with current regulations and guidelines. Blood samples were collected from the heart and aorta, allowed to clot at room temperature, and centrifuged to separate serum, which was stored at −80 °C until biochemical analyses.

Hearts were excised by transecting the massive vessels and processed for histopathological evaluation using standard techniques. Characteristic morphological changes were documented through colour microphotography. All histological analyses were performed in a blinded manner.

### 2.3. Histopathological Analysis

Cardiac tissue samples were processed using standard histological procedures and stained with Masson’s trichrome for morphological assessment of myocardial architecture and connective tissue deposition. Histopathological evaluation included assessment of replacement fibrosis, defined as areas of cardiomyocyte loss replaced by collagen fibers; interstitial fibrosis, defined as increased connective tissue deposition between cardiomyocytes; and perivascular fibrosis, defined as increased connective tissue accumulation surrounding myocardial blood vessels. Representative myocardial sections were evaluated at ×100 magnification (Nikon Corporation microscope, Tokyo, Japan).

In addition to qualitative histopathological assessment, quantitative stereological analysis was performed to determine the volume densities of the principal myocardial tissue compartments. The following stereological parameters were evaluated: VGPV, representing the volume density of the perivascular space; VGI, representing the volume density of the myocardial interstitium; VGV, representing the volume density of connective tissue; and VGK, representing the volume density of cardiomyocytes. Volume densities were determined using a stereological point-counting approach and expressed as the proportion of the analyzed myocardial volume occupied by the corresponding tissue compartment. All histological and stereological analyses were performed in a blinded manner according to the previously described methodological approach [32].

### 2.4. Biochemical Analyses

Biochemical measurements were performed using standard laboratory procedures with commercially available diagnostic kits (Abcam, Cambridge, UK and Biorbyt Ltd., Cambridge, UK), either colorimetrically or by enzyme-linked immunosorbent assay (ELISA), following the manufacturers’ protocols. These analyses were conducted to assess the effects of the applied therapeutic interventions and to evaluate key biomarkers of inflammation and oxidative stress, providing insight into the underlying pathophysiological mechanisms.

sST2 Determination: Serum sST2 was quantified using a sandwich ELISA. Microtiter plates coated with ST2-specific antibodies were incubated with standards and samples, followed by an antibody cocktail, washing, and TMB substrate development. The reaction was stopped with stop solution, and absorbance was measured at 600 nm using a STAT FAX 2100 microplate reader (Awareness Technology, Palm City, FL, USA). The standard range was 70.31–4500 pg/mL, with a detection limit of 5.36 pg/mL.

IL-33 Determination: Serum IL-33 levels were determined using a similar sandwich ELISA protocol. Absorbance was measured at 600 nm. The standard range was 46.9–3000 pg/mL, with a detection limit of 6 pg/mL.

SOD Activity: Serum superoxide dismutase (SOD) activity was measured using a commercially available Superoxide Dismutase Activity Assay Kit (Colorimetric; Abcam, Cambridge, UK; catalog no. ab65354), based on the WST-1/xanthine oxidase method. Superoxide anions generated by xanthine oxidase react with WST-1 to form a water-soluble formazan dye, while SOD inhibits this reaction. SOD activity was expressed as the percentage inhibition rate (% inhibition), calculated according to the manufacturer’s instructions. Malondialdehyde (MDA) levels were assessed using a commercially available Rat MDA ELISA Kit (Biorbyt Ltd., Cambridge, UK; catalog no. orb782406), based on a competitive inhibition immunoassay. After incubation with MDA-specific antibodies and a biotinylated conjugate, streptavidin-HRP and substrate were added. Absorbance, inversely proportional to MDA concentration, was measured at 450 nm. The standard range was 15.63–1000 pg/mL, with a detection limit of 5.39 pg/mL. MDA concentrations were calculated according to the manufacturer’s instructions and expressed as pg/mL.

### 2.5. Statistical Analysis

The data were analysed using SPSS software (version 23.0). The Shapiro–Wilk test assessed data normality. Continuous variables with normal distribution were expressed as mean ± SD; non-normally distributed variables as median and interquartile range; and categorical variables as absolute and relative frequencies. Group comparisons were performed using the Student’s *t*-test for two groups and one-way ANOVA followed by Bonferroni post-hoc testing for three or more groups. Non-parametric data were analysed using the Mann–Whitney U or Kruskal–Wallis tests as appropriate. Categorical variables were compared using the chi-square or Fisher’s exact test. A *p*-value < 0.05 was considered statistically significant.

## 3. Results

### 3.1. Biochemical Findings

The biochemical findings across the experimental groups are summarized in Table 1.

Superoxide dismutase (SOD) activity in the control group was 88.4 ± 2.3, while higher values were observed in the DM group (95.1 ± 1.9). The NAC group showed significantly lower SOD activity compared to both the control and DM groups (71.17 ± 6.3; *p* < 0.05). The glycine group also demonstrated reduced SOD activity compared to the control and DM groups (84.5 ± 4.5; *p* < 0.05), but significantly higher values than the NAC group (*p* < 0.05). The lowest SOD activity was observed in the NAC + glycine group (71.6 ± 8.0), which was significantly lower compared to all other groups (*p* < 0.05). Overall, differences between groups were statistically significant (*p* < 0.0001).

Malondialdehyde (MDA) concentration in the control group was 89.0 ± 16.6, while a markedly higher level was observed in the DM group (314.0 ± 111.2; *p* < 0.05). All treatment groups exhibited significantly lower MDA levels compared to the DM group (*p* < 0.05). The lowest values were found in the NAC group (69.4 ± 12.8), whereas the NAC + glycine group showed significantly higher levels compared to NAC (100.6 ± 2.9; *p* < 0.05). Differences between groups were statistically significant (*p* < 0.0001).

Soluble ST2 concentrations in the control group were 320.0 ± 53.1, while significantly higher levels were observed in the DM group (709.1 ± 43.5; *p* < 0.05). All treatment groups had significantly lower ST2 levels compared to the DM group (*p* < 0.05). The glycine group showed higher sST2 levels compared to both the control and NAC groups (525.3 ± 31.8; *p* < 0.05), whereas the NAC + glycine group exhibited the lowest sST2 levels (286.9 ± 60.5), significantly lower than those in the glycine group (*p* < 0.05). Differences between groups were statistically significant (*p* < 0.0001).

Interleukin-33 (IL-33) concentration in the control group was 186.8 ± 28.5, while significantly higher levels were observed in the DM group (651.9 ± 124.4; *p* < 0.05). All treatment groups demonstrated significantly lower IL-33 levels compared to the DM group (*p* < 0.05). The glycine group had higher IL-33 levels compared to both the control and NAC groups (278.7 ± 27.3; *p* < 0.05), whereas the NAC + glycine group showed the lowest levels (150.3 ± 22.5), significantly lower than those in the NAC group (*p* < 0.05). Differences between groups were statistically significant (*p* < 0.0001).

### 3.2. Morphological Findings

The stereological parameters of myocardial remodeling across the experimental groups are presented in Table 2.

VGPV in the control group was 0.30 ± 0.01, while significantly higher values were observed in the DM group (0.36 ± 0.01; *p* < 0.05). All treatment groups (NAC, glycine, and NAC + glycine) demonstrated significantly lower VGPV compared to the DM group (*p* < 0.05), with values comparable to the control group. Overall, differences between groups were statistically significant (*p* < 0.0001).

VGI was 0.09 ± 0.005 in the control group and significantly increased in the DM group (0.14 ± 0.01; *p* < 0.05). The NAC group showed intermediate values (0.11 ± 0.004), significantly lower than the DM group but still higher compared to the control group (*p* < 0.05). In contrast, both the glycine and NAC + glycine groups had significantly lower VGI compared to both the DM and NAC groups (0.09 ± 0.003; *p* < 0.05), reaching values similar to the control group. Differences between groups were statistically significant (*p* < 0.0001).

VGV was lowest in the control group (0.02 ± 0.002) and significantly increased in the DM group (0.05 ± 0.01; *p* < 0.05). All treatment groups showed significantly reduced VGV compared to the DM group (*p* < 0.05). The glycine group demonstrated values comparable to the control group (0.02 ± 0.002), while the NAC and NAC + glycine groups had slightly higher but still significantly reduced values (0.03 ± 0.002 and 0.03 ± 0.004, respectively). Overall differences were statistically significant (*p* < 0.0001).

VGK was 0.90 ± 0.007 in the control group and significantly decreased in the DM group (0.84 ± 0.008; *p* < 0.05). All treatment groups demonstrated significantly higher VGK compared to the DM group (*p* < 0.05), with values approaching or exceeding those of the control group. The highest values were observed in the NAC + glycine group (0.913 ± 0.017), which was significantly higher compared to the NAC group (*p* < 0.05). Differences between groups were statistically significant (*p* < 0.0001).

### 3.3. Pathohistological Findings

**Figure 1:**
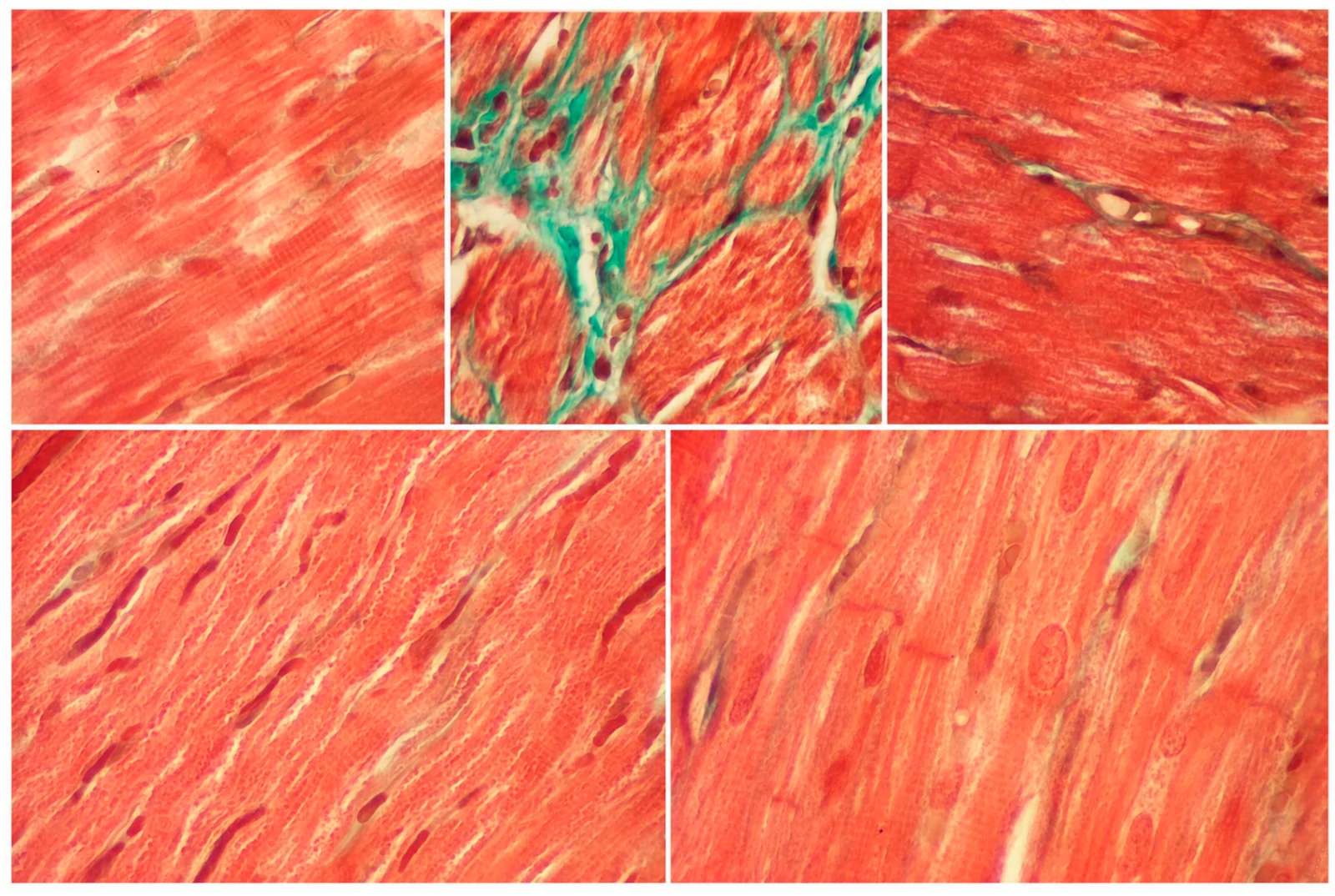
Representative histological sections of myocardial tissue (Masson’s trichrome, ×100). (**Top row**) Left—regularly arranged cardiomyocytes with preserved morphology, interwoven with a small amount of interstitial connective tissue (control group); Middle—replacement fibrosis (diabetic group); Right—mildly expanded interstitial space with areas of more prominent connective tissue (NAC group). (**Bottom row**) Left—interstitial space with loosely organized connective tissue and focal accumulation of ground substance (Glycine group); Right—preserved cardiomyocytes with clearly visible intercalated discs (NAC + Glycine group).

Representative histopathological findings across the experimental groups are shown in Figure 1.

The image in the top row on the left, corresponding to the control group, demonstrates well-preserved myocardial architecture, characterized by regularly arranged cardiomyocytes forming a network with intact cross-striations and clearly defined intercalated discs. The cells exhibit homogeneous eosinophilic sarcoplasm with orderly organized myofibrils and centrally located nuclei with finely granular chromatin, while occasional binucleated cardiomyocytes are also observed. The myocardial fibers are embedded within a loose endomysial interstitium containing capillaries, with focal areas of increased ground substance. Cardiomyocytes show variable orientation, predominantly forming layers and bundles separated by delicate connective tissue. In certain regions, interstitial connective tissue merges into wider spaces associated with larger vascular structures, while a thin layer of denser, well-organized perivascular connective tissue is evident around arteries and arterioles.

The image in the top row in the middle, corresponding to the diabetic group, demonstrates marked structural alterations of the myocardium. Cardiomyocytes appear irregularly arranged, with disrupted cross-striations and features of vacuolization, necrosis, and focal atrophy. The cytoplasm shows increased eosinophilia with disorganized myofibrillar architecture, while nuclei exhibit altered staining characteristics ranging from hyperchromatic to pyknotic. The interstitial space is expanded, with increased deposition of connective tissue, including areas of dense, compact collagen, accompanied by dilated and hyperemic microvasculature. In some regions, degenerated cardiomyocytes are replaced by organized fibrotic tissue. Additionally, small and medium-sized arteries show prominent endothelium and subendothelial connective tissue, while larger vessels and arterioles are surrounded by a thickened layer of dense connective tissue merging with the adventitia.

The image in the top row on the right, corresponding to the NAC group, shows mildly altered myocardial architecture. Some cardiomyocytes appear morphologically changed, with increased eosinophilia and darker nuclei. The interstitial space is slightly expanded, containing moderately increased connective tissue, within which the vascular component is locally more prominent. Perivascular connective tissue surrounding arteries and arterioles appears more pronounced than usual and is less clearly demarcated from the adventitial connective tissue.

The image in the bottom row on the left, corresponding to the glycine group, shows relatively preserved myocardial structure. Cardiomyocytes display mild alterations in morphology and arrangement, while the interstitium contains delicate connective tissue with locally more prominent microvasculature. The periarterial and periarteriolar connective tissue is mildly accentuated.

The image in the bottom row on the right, corresponding to the NAC + glycine group, demonstrates relatively preserved myocardial architecture. Cardiomyocytes are regularly arranged, with intact intercalated discs and preserved cytoplasmic striations. The muscle cells are surrounded by a thin, uniform layer of endomysial connective tissue. A mildly accentuated connective tissue sheath is observed around arterial vessels.

## 4. Discussion

A complicated interaction between metabolic dysregulation, oxidative stress, inflammation, and progressive cardiac remodelling is what differentiates DCM from other forms of myocardial remodelling. A comprehensive evaluation of these interconnected pathways is presented in the current study. The findings indicate that diabetes causes significant modifications in biochemical, molecular, and histological parameters. On the opposing side, treatment with NAC and glycine, particularly in combination, has been shown to exert significant cardioprotective effects [1,2,3,4].

The current model demonstrated that diabetes correlated with a significant elevation in oxidative stress and inflammation indicators, as well as structural changes in cardiac tissue. The findings align with established evidence indicating that compromised glucose utilisation and a transition to free fatty acid metabolism result in mitochondrial dysfunction, increased ROS production, and the activation of pro-inflammatory signalling pathways, as shown by Chong et al. [33]. These disruptions lead to maladaptive cardiac remodelling via pathways that include lipid buildup, protein kinase C activation, and disruption of cellular homeostasis.

Simultaneously, cardiac injury generated by diabetes is exacerbated by increased apoptosis and dysregulated autophagy. Although autophagy may initially provide protective benefits, its prolonged activation leads to structural degradation, extracellular matrix growth, and advancing fibrosis. These mechanisms are corroborated by the research of Eguchi et al. [34] and Zayas-Arrabal et al. [35], which illustrated the pivotal involvement of inflammation and oxidative stress in the arrhythmogenic and structural remodelling of the diabetic myocardium. It is crucial to note that therapeutic intervention with NAC and glycine achieved a consistent attenuation of these pathological processes, as demonstrated by decreases in oxidative stress markers, inflammatory mediators, and histological indicators of myocardial injury. The magnitude of treatment effects varied across the assessed outcomes. The combined treatment showed particularly favorable effects on circulating sST2 and IL-33 levels and several structural parameters, whereas some oxidative stress outcomes showed a greater response to NAC monotherapy. This pattern may reflect complementary, but outcome-dependent, effects of glycine and NAC. Based on previous experimental evidence, these effects may involve mechanisms including restoration of intracellular glutathione levels, modulation of AGE/RAGE signalling, and improvement of mitochondrial function; however, these mechanisms were not directly investigated in the present study [36].

The diabetic group exhibited a significant increase in soluble ST2 (sST2), which serves as a critical biomarker of myocardial stress, inflammation, and remodelling. This finding represents one of the most prominent biomarker changes observed in the present study. As evidenced by the research conducted by Lin et al. [37] and Fousteris et al. [38], elevated sST2 levels have been consistently associated with adverse cardiovascular outcomes, such as the progression of heart failure and an increase in mortality. In addition to its function as a biomarker, sST2 is a critical integrative indicator of cardiac remodelling, as it reflects underlying myocardial stress, inflammation, and fibrotic activity. It is crucial to note that elevated sST2 levels have been demonstrated to offer prognostic information that is distinct from that of conventional biomarkers, thereby emphasising its clinical significance in the context of disease monitoring and risk stratification. Mechanically, sST2 functions as a decoy receptor for IL-33, which inhibits the cardioprotective IL-33/ST2L signalling axis, thereby promoting apoptosis, hypertrophy, and fibrosis [39,40].

The observed decrease in circulating sST2 levels after NAC and glycine treatment, particularly in the combination group, indicates a treatment-associated change in this biomarker. Although this finding may suggest an association with the IL-33/ST2 system, direct modulation of myocardial IL-33/ST2 signalling cannot be established from circulating biomarker concentrations alone. As circulating sST2 is associated with oxidative stress, inflammation, and myocardial remodeling, its reduction may reflect a more favorable biomarker profile; however, it should not be interpreted as direct evidence of attenuation of these underlying pathogenic processes. The observed changes in sST2 were accompanied by alterations in the measured oxidative stress markers, supporting an association between these biochemical changes in the present experimental model. Diabetes correlated with a significant elevation in malondialdehyde (MDA), indicating increased lipid peroxidation and oxidative injury. MDA significantly enhances pro-inflammatory effects, intensifying cytokine signalling and facilitating myocardial remodelling [41,42,43,44,45].

Treatment with NAC and glycine markedly decreased MDA levels, although the magnitude of this effect differed between treatment groups, with the lowest MDA levels observed in the NAC monotherapy group. These effects may potentially involve complementary mechanisms, including modulation of AGE/RAGE signaling by glycine and antioxidant and cardioprotective effects of NAC, as suggested by previous experimental evidence [46,47]. In the diabetic group, there was a compensatory increase in SOD activity, which reflects an adaptive response to the elevated levels of ROS. Treatment resulted in a reduction in SOD activity alongside a marked decrease in MDA levels. This pattern may reflect a reduced requirement for compensatory SOD activity following attenuation of the oxidative burden rather than suppression of antioxidant defense; however, this interpretation remains tentative in the absence of additional redox biomarkers [48,49,50].

The oxidative alterations were accompanied by changes in circulating IL-33 and sST2 levels. The increased circulating IL-33 levels observed in the diabetic group may reflect a compensatory response associated with diabetes-related myocardial stress; however, the functional significance of this finding cannot be determined from circulating IL-33 and sST2 concentrations alone. The concomitant changes in circulating IL-33 and sST2 levels suggest an association with the IL-33/ST2 system; however, they cannot establish direct modulation of myocardial IL-33/ST2 signaling, as ST2L expression and downstream signaling components were not assessed. Importantly, IL-33 exerts context-dependent effects in cardiovascular biology, including cardioprotective actions through ST2L; therefore, the observed reduction in circulating IL-33 should not be interpreted independently as evidence of therapeutic benefit [40,51].

Histological findings further support these biochemical and molecular observations. Diabetes induced expansion of interstitial and perivascular spaces, increased connective tissue deposition, and reduced cardiomyocyte density, reflecting myocardial fibrosis and extracellular matrix remodeling [52,53].

Treatment with NAC and glycine, administered either alone or in combination, attenuated these structural alterations, although the magnitude of the observed effects varied across the assessed stereological parameters. Both agents preserved myocardial architecture while decreasing interstitial expansion and connective tissue accumulation. These results are consistent with antifibrotic mechanisms that involve the inhibition of collagen synthesis and fibroblast activation [54].

Preservation of cardiomyocyte volume density indicates attenuation of diabetes-associated structural myocardial alterations; however, it cannot be interpreted as direct evidence of reduced cardiomyocyte apoptosis. The favorable effects observed with combined NAC and glycine treatment may reflect complementary mechanisms, although the magnitude of benefit varied across the assessed biochemical and structural outcomes. The central role of sST2 observed in this study is consistent with its established significance as a prognostic biomarker in heart failure, as seen from a broader clinical perspective. Negative consequences are linked to elevated sST2 levels, regardless of conventional biomarkers. The IL-33/sST2 balance is becoming more widely acknowledged as a determinant of cardiac remodelling outcomes. This is especially pertinent in diabetic populations that are characterised by chronic inflammation [55,56].

Sekhar et al. [57] have reported that the concept of combined NAC and glycine therapy (GlyNAC) is being increasingly supported by emergent evidence that demonstrates significant improvements in oxidative stress, mitochondrial function, and overall metabolic health. In addition to these systemic effects, GlyNAC has been demonstrated to improve redox homeostasis and increase intracellular glutathione availability, thereby targeting critical mechanisms related to metabolic and cardiovascular dysfunction. The therapeutic potential of combined antioxidant strategies in modulating oxidative stress-driven myocardial remodelling is further reinforced by these findings, which are closely aligned with the results of the present study [55,56].

Additionally, a unified therapeutic approach is achieved by concurrently targeting oxidative stress, inflammation, and fibrosis. NAC and glycine are particularly well-suited for complex conditions like DCM due to their ability to act at multiple levels of disease progression.

Several limitations of this study should be acknowledged. First, the study was conducted using an experimental animal model, and therefore the findings cannot be directly extrapolated to human diabetic cardiomyopathy. Second, the relatively limited and unequal group sizes (6–8 per group) should be considered when interpreting the findings. A formal a priori power analysis was not performed, and the sample size was determined within the practical constraints of the experimental design and the principle of minimizing the number of animals used. Consequently, the study may have had limited statistical power to detect smaller between-group differences. Third, the 12-week observation period may restrict the assessment of longer-term myocardial remodeling. Dedicated small-animal echocardiography was not available in our laboratory, precluding serial functional assessment of cardiac structure and function during the development of diabetes-associated myocardial remodeling. Consequently, the experimental duration and expected time course of myocardial fibrosis and structural remodeling were based on previously published and validated streptozotocin-induced diabetic rat models. Nevertheless, myocardial remodeling was directly confirmed at the end of the experiment by histopathological and stereological analyses.

Additional limitations relate to the biochemical and morphological assessments. Oxidative stress assessment was limited to MDA and SOD, while additional redox biomarkers such as GSH, CAT, and superoxide were not directly measured. Furthermore, glycemic control was not longitudinally assessed during the 12-week treatment period, and HbA1c was not measured; therefore, a potential contribution of treatment-related changes in glycemic status to the observed biochemical and cardiac structural effects cannot be excluded. Although the stereological assessment was performed on randomly selected microscopic fields by a blinded investigator, the exact number of fields analyzed per specimen was not prospectively recorded, which represents a methodological limitation of the study. In addition, the histopathological assessment of myocardial fibrosis was semiquantitative and therefore may be subject to observer-dependent variability, despite blinded evaluation.

Future studies incorporating adequately powered experimental groups, serial echocardiographic assessment, longitudinal evaluation of glycemic control, a broader panel of oxidative stress biomarkers, and standardized prospective documentation of stereological sampling would provide a more comprehensive assessment of the temporal relationship between metabolic alterations, oxidative stress, myocardial fibrosis, and cardiac dysfunction. Accordingly, the present findings should primarily be interpreted as evidence of biochemical and structural myocardial remodeling rather than functional diabetic cardiomyopathy.

In summary, NAC and glycine, administered either alone or in combination, attenuated several biochemical and structural alterations associated with experimental diabetes. The magnitude of these effects varied across the assessed outcomes, supporting potentially complementary, but outcome-dependent, effects of these interventions. These findings warrant further investigation of NAC- and glycine-based approaches in diabetes-associated myocardial remodeling.

## 5. Conclusions

This study demonstrates that streptozotocin-induced diabetes is associated with alterations in oxidative stress and inflammatory biomarkers, accompanied by significant structural myocardial remodeling. These findings provide a biochemical and morphological basis for evaluating the effects of targeted antioxidant interventions on diabetes-associated myocardial alterations.

A significant discovery is the pivotal function of sST2 as a comprehensive measure of cardiac stress, connecting oxidative and inflammatory mechanisms with structural remodelling. The observed changes in circulating IL-33 and sST2 concentrations further support their potential relevance as biomarkers of diabetes-associated myocardial remodeling.

The administration of NAC and glycine, either alone or in combination, attenuated several biochemical and structural alterations associated with diabetes. The magnitude of these effects varied across the assessed outcomes, with the combined regimen showing particularly favorable effects on circulating sST2 and IL-33 levels and selected indices of myocardial remodeling, whereas some oxidative stress parameters showed a greater response to NAC monotherapy. This pattern suggests potentially complementary, but outcome-dependent, effects of NAC and glycine rather than uniform superiority of the combined regimen. Notwithstanding the constraints of the experimental paradigm, these findings support further investigation of NAC- and glycine-based interventions for limiting diabetes-associated cardiac remodeling, including studies aimed at clarifying the mechanisms underlying their individual and combined effects.

## Figures and Tables

**Table 1:** Serum Biochemical Parameters Across Experimental Groups.

	Groups					*p*
Variable	Control	DM	NAC	Glycine	NAC + Glycine	
SOD (% of inhibition)	88.4 ± 2.3	95.1 ± 1.9	71.17 ± 6.3 **	84.5 ± 4.5 **,#	71.6 ± 8.0 *,**,†	*p* < 0.0001
MDA (pg/mL)	89.0 ± 16.6	314.0 ± 111.2 *	69.4 ± 12.8 **	79.1 ± 18.9 **	100.6 ± 2.9 **,#	*p* < 0.0001
sST2 (pg/mL)	320.0 ± 53.1	709.1 ± 43.5 *	341.4 ± 33.4 **	525.3 ± 31.8 *,**,#	286.9 ± 60.5 **,†	*p* < 0.0001
IL-33 (pg/mL)	186.8 ± 28.5	651.9 ± 124.4 *	180.6 ± 11.9 **	278.7 ± 27.3 **	150.3 ± 22.5 **,#	*p* < 0.0001

Control—control group; DM—diabetes mellitus group; NAC—N-acetylcysteine for 12 weeks; glycine—glycine for 12 weeks; NAC + Glycine—combined N-acetylcysteine and glycine for 12 weeks; overall group differences *p* < 0.0001. Symbols indicate statistically significant differences between groups: * vs. control; ** vs. DM; # vs. NAC; † vs. glycine.

**Table 2:** Stereological Parameters of Myocardial Remodeling Across Experimental Groups.

	Groups					*p*
Variable	Control	DM	NAC	Glycine	NAC + Glycine	
VGPV	0.30 ± 0.01	0.36 ± 0.01 *	0.30 ± 0.01 **	0.30 ± 0.01 **	0.30 ± 0.008 **	*p* < 0.0001
VGI	0.09 ± 0.005	0.14 ± 0.01 *	0.11 ± 0.004 *,**	0.09 ± 0.003 **,#	0.09 ± 0.003 **,#	*p* < 0.0001
VGV	0.02 ± 0.002	0.05 ± 0.01 *	0.03 ± 0.002 *,**	0.02 ± 0.002 **	0.03 ± 0.004 **	*p* < 0.0001
VGK	0.90 ± 0.007	0.84 ± 0.008 *	0.89 ± 0.005 **	0.90 ± 0.016 **	0.913 ± 0.017 **,#	*p* < 0.0001

Control—control group; DM—diabetes mellitus group; NAC—animals treated with N-acetylcysteine for 12 weeks; glycine—animals treated with glycine for 12 weeks; NAC + Glycine—animals treated with combined N-acetylcysteine and glycine for 12 weeks; overall group differences *p* < 0.0001. Symbols indicate statistically significant differences between groups: * vs. Control; ** vs. DM; # vs. NAC.

## Data Availability

The data are not publicly available due to institutional and ethical restrictions but are available from the corresponding author upon reasonable request.
